# Identification and Prioritization of Relationships between Environmental Stressors and Adverse Human Health Impacts

**DOI:** 10.1289/ehp.1409138

**Published:** 2015-04-10

**Authors:** Shannon M. Bell, Stephen W. Edwards

**Affiliations:** 1 Oak Ridge Institute for Science and Education, Oak Ridge, Tennessee, USA; 2 Integrated Systems Toxicology Division, National Health and Environmental Effects Research Laboratory, Office of Research and Development, U.S. Environmental Protection Agency, Research Triangle Park, North Carolina, USA

## Abstract

**Background:**

There are > 80,000 chemicals in commerce with few data available describing their impacts on human health. Biomonitoring surveys, such as the NHANES (National Health and Nutrition Examination Survey), offer one route to identifying possible relationships between environmental chemicals and health impacts, but sparse data and the complexity of traditional models make it difficult to leverage effectively.

**Objective:**

We describe a workflow to efficiently and comprehensively evaluate and prioritize chemical–health impact relationships from the NHANES biomonitoring survey studies.

**Methods:**

Using a frequent itemset mining (FIM) approach, we identified relationships between chemicals and health biomarkers and diseases.

**Results:**

The FIM method identified 7,848 relationships between 219 chemicals and 93 health outcomes/biomarkers. Two case studies used to evaluate the FIM rankings demonstrate that the FIM approach is able to identify published relationships. Because the relationships are derived from the vast majority of the chemicals monitored by NHANES, the resulting list of associations is appropriate for evaluating results from targeted data mining or identifying novel candidate relationships for more detailed investigation.

**Conclusions:**

Because of the computational efficiency of the FIM method, all chemicals and health effects can be considered in a single analysis. The resulting list provides a comprehensive summary of the chemical/health co-occurrences from NHANES that are higher than expected by chance. This information enables ranking and prioritization on chemicals or health effects of interest for evaluation of published results and design of future studies.

**Citation:**

Bell SM, Edwards SW. 2015. Identification and prioritization of relationships between environmental stressors and adverse human health impacts. Environ Health Perspect 123:1193–1199; http://dx.doi.org/10.1289/ehp.1409138

## Introduction

There are very few human health or exposure data for the majority of the > 80,000 chemicals in commerce (Egeghy et al. 2012; Judson et al. 2009). The lack of data poses challenges to those looking to mitigate the potential risks or evaluate impacts in a comprehensive manner. The National Health and Nutrition Examination Survey (NHANES) [Centers for Disease Control and Prevention National Center for Health Statistics (CDC NCHS) 2010] provides a snapshot of the current health status of a representative U.S. population. Numerous studies using the NHANES and similar data sets have been used to extract possible associations between markers of exposure to environmental chemicals and possible health effects (Patel and Ioannidis 2014). The nature of the data sets and the models used makes it a challenge to compare the studies in a systematic way, and consequently leads to an iterative process involving multiple individual hypotheses being tested over the course of the analysis (Patel and Ioannidis 2014). This results in a complicated design in which it is impossible to account for multiple individual *a priori* hypothesis tests (Patel and Ioannidis 2014). The consequence of this is more false positive relationships and an overall lack of transparency.

Researchers have conducted large-scale analyses of the data sets (Gennings et al. 2012; Liu et al. 2009; Patel et al. 2010, 2012a, 2012b), enabling better control for the multiple testing effects of running several regression models. Patel et al. (2010, 2012a, 2012b) used FDR (false discovery rate) correction in a semi-supervised approach to test hundreds of regression models associating environmental factors with a specific disease outcome in what they coined “environment-wide association study” (EWAS). This approach enables testing of factors that may not be implicated in other work as having a relationship with the outcome, increasing the likelihood that new hypotheses are generated. It also makes results more comparable with traditional approaches, which may be advantageous when aggregating results of several studies. Another approach has been to lump variables, combining compounds in a similar class or affecting the same pathway. This lumping approach helps to limit the number of tests run and can provide additional insight on how effects might be related. Liu et al. (2009) looked at functionally related chemicals and their effects on the liver by first prioritizing the chemicals of strongest effect based on canonical correlation before building regression models. Gennings et al. (2012) went a step further in defining agglomerative markers for both health outcomes and environmental chemicals. The process of calculating a relative weight for the chemicals in a group enables identification of the ones having the most effect on the outcome, and can help prioritize or identify additional confounding variables for individual regression models. Because one challenge is in defining and assigning the negative health outcomes, combined outcomes such as general wellness may facilitate model development when an exact association is still unclear.

Unfortunately, none of these approaches address the challenges of missing/sparse data and identifying possible confounding variables in instances where there is no co-occurrence data. Additionally, the regression models typically used are impractical for a comprehensive survey of all compounds versus all relevant health measures in the NHANES data. To address these issues as well as to enable prioritization of associations, we developed a workflow based on frequent itemset mining (FIM) (Bell and Edwards 2014). This approach enables consuming a data set and generating associations that *a*) describe the relative likelihood of an exposure and health event not co-occurring by chance, *b*) enable relative ranking for prioritization even in the absence of co-occurrence, and *c*) are generated by a simple, transparent format for communication with subject domain experts.

Here we present the application of the FIM approach to a meta-analysis of the 1999–2010 NHANES cycles. This work aims to address the ability of a market basket approach to facilitate prioritization of chemical/health associations, comparisons and reconciliation of prior published works that consider only a subset of the data, and hypothesis generation for follow-up studies. Exploration of the robustness of the approach to the different data cycles and confounding variables is shown. This approach was evaluated via two case studies, the C8 Science Panel’s review of perfluorooctanoic acid (PFOA) (C8 Science Panel 2005) and a comparison with the multiple regression approach presented in the study by Patel et al. (2010) looking at type 2 diabetes. Given the positive results from this evaluation, we propose the use of this FIM workflow to assist in interpreting the literature and prioritizing chemicals and chemical–health associations for further study.

## Methods

**Figure 1:**
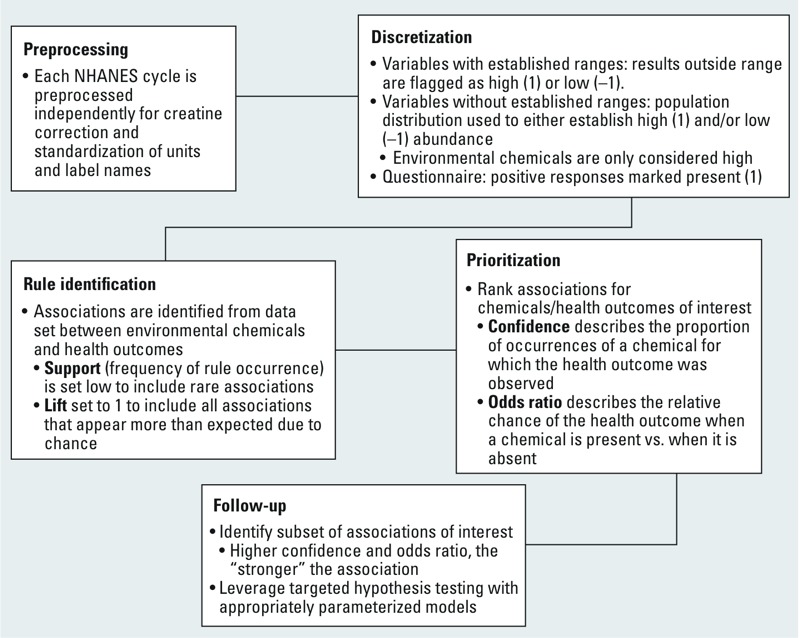
General workflow using FIM. A brief overview of the workflow from Bell and Edwards (2014) is described. Values used in the Discretization step are a key determinant of the generated rules and should be reviewed.

*Data sources and processing*. Figure 1 outlines the general workflow for the generation and use of associations between chemical and effects markers based on NHANES data. Data from NHANES 1999–2010 (CDC NCHS 2010) form the basis of this study. For details on the specific variables used, including the variable label and a description, see Supplemental Material, File S1. For more information on the variable distribution and cutoffs used for discretization (along with source), see Supplemental Material, File S2. Variables were classified as environmental chemicals (E), health biomarkers (H), or questionnaire responses (Q). Of the 373 possible unique variables, the data contained 236 markers for environmental chemicals, 104 of health biomarkers, and 33 questionnaire responses. Across all cycles only 28, 52, and 24 measures for E, H, and Q, respectively, were in all the data sets. Data processing and analysis followed the approach described by Bell and Edwards (2014). Urinary measures for E and H were creatinine corrected due to the high level of correlation between measures in the absence of the correction (Figure 1, Preprocessing). Discretization (Figure 1, Discretization) was done by identifying values below (if applicable), at, or above (–1, 0, 1, respectively) normal, which was defined either by the population distribution (< 2.5 percentile or > 97.5 percentile) or clinically established values (outside of predefined normal range). For variables having unclear clinical ranges (such as some vitamin and carotenoids), population distribution was used. All values used for the discretization are recorded in the Supplemental Material, File S2, along with references for those values derived from clinically established values. Full text descriptions of the variables can be found in Supplemental Material, File S1. Questionnaire data were taken as yes (1) if there was a positive response, no (0) or no data (NA) otherwise. Two variables were derived from the questionnaire responses. Grade 1 angina, abbreviated here as “CDQ99,” is defined based on the answers to questions relating to chest pain as specified by the CDC (http://www.nber.org/nhanes/2005_2006/downloads/cdq_d.pdf). Cardiovascular disease, abbreviated here as “CD,” is defined based on grade 1 angina, self-reported angina, or heart attack (i.e., CDQ99 = 1 or MCQ160D = 1 or MCQ160E = 1 or MCQ160C = 1). A large portion of the population has diabetes or has not been diagnosed as diabetic but the laboratory tests indicate a risk (high blood glucose and hemoglobin A1c); therefore, a marker for diabetes (Dia) was added to include both these groups (Dia = DIQ010 = 1 or LBXGLU = 1 or LBXGH = 1).

*Identification of associations*. Associations (Figure 1, Rule identification) were identified as described in Bell and Edwards (2014) using the FIM approach [see Supplemental Material, Files S3 (text version) and S4 (Excel version, “Spreadsheet”)]. FIM [also known as market basket analysis or association rule mining (Agrawal et al. 1993; Borgelt and Kruse 2002; Borgelt 2003, 2012; Hahsler et al. 2005)] looks for patterns of frequently co-occurring items within a data set. Interest measures (Hahsler et al. 2005; Tan et al. 2004) are used to describe the likelihood of an itemset or an association rule (X → Y). “Support” describes the proportion of the transactions (samples, subjects) containing the rule (X and Y), whereas “confidence” of a rule is the proportion of all transactions having X that also have Y. Stated another way: the probability of finding Y given X. “Lift” measures how frequently X and Y co-occur versus the expectation that they were independent. Odds ratio relates the “risk” of Y when X is present relative to when X is not present.

A minimum support value of 10 samples was used in conjunction with a minimum confidence of 0.1 and lift of 1.0 to account for the sparsity of data. For a more detailed discussion of setting confidence and support measures, see Hahsler et al. (2005) and Megiddo and Srikant (1998). Rules were identified with the chemical as the antecedent (Bell and Edwards 2014). Lift interest measure (Brin et al. 1997) from the association rules was used as a basis for rank comparison across NHANES cycles and data subsets. For the rank-based comparisons to look at the impact of cycle years or confounding variables on the rules generated, the top association (by highest lift) was numbered 1. Associations not found in that particular data set were assigned a value greater than the largest number of associations for the itemsets compared to include them in the ranking. All analyses were done in R (version 3.0.3) (R Core Team 2013) using the arules package (Hahsler et al. 2005) for generating the association rules. All code and input data required to replicate this study are available in the Supplemental Material, File S5, “Zip.”

*Case studies*. We compared the list of factors examined by the C8 Science Panel (2005) with the variable list used in the present study to identify common outcomes considered. All associations containing PFOA and relating to a health outcome from the questionnaire data were extracted from Supplemental Material, File S3, for consideration (see also Supplemental Material, File S4, Worksheet “StrongPFOA”). For any outcome where either the C8 panel found a probable association or the FIM yielded an association with PFOA, the details from the C8 panel were considered for potential sources of discrepancy to gauge whether there was a likely false positive or false negative from the FIM. For comparison with the EWAS study of diabetes by Patel et al. (2010), associations including individuals with elevated fasting blood glucose (LBXGLU = 1; see Supplemental Material, File S2), and those who had a positive diabetes aggregate marker (Dia, described above) were considered. The list of significant associations taken from Patel et al. (2010) were used to compare with the FIM. FIM associations covered NHANES cycles 1999–2010, whereas the ones in the EWAS study covered each cycle from 1999 through 2006 individually.

## Results

**Figure 2:**
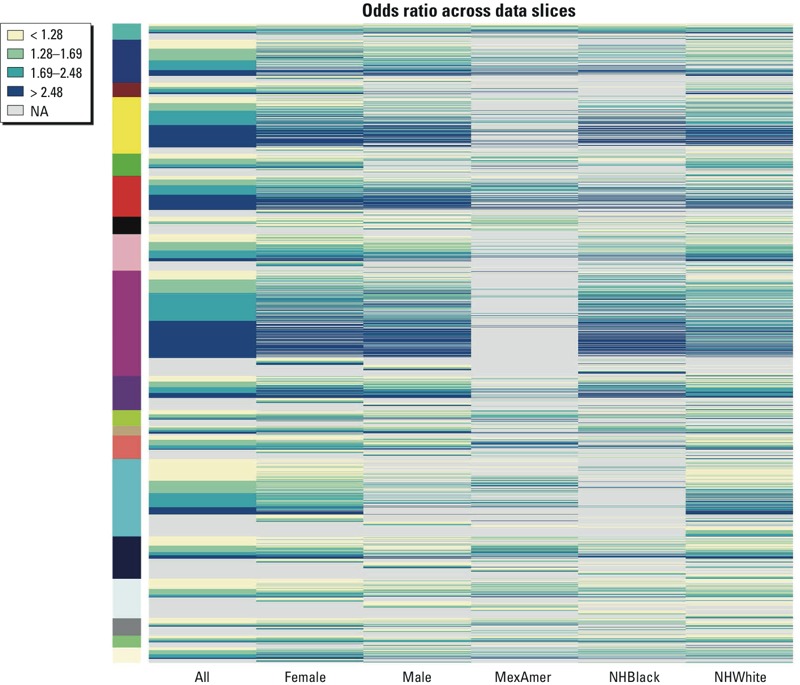
Heat map of associations across the different slices. Colors are based on odds ratio of the associations, associations not found or below the threshold are in gray (NA). Abbreviations: MexAmer, Mexican American; NHBlack, non-Hispanic black; NHWhite, non-Hispanic white. Color labels on the left highlight the different chemical groups for the association. From bottom to top: Light yellow = drinking water volatile organic compounds; light green = urinary perchlorate, nitrate, and thiocyanate; gray = phytoestrogens; light cyan = phthalates; midnight blue = urinary metals; cyan = current use pesticides; salmon = urinary arsenic; tan = smoking; green yellow = environmental pesticides; purple = PFC (perfluorinated compound); magenta = PCB; pink = PAH; black = organophosphate pesticides; red = organochlorine pesticides; green = environmental phenols; yellow = DFP (diisopropyl fluorophosphate); brown = carbamates; blue = blood volatile organic compounds; turquoise = blood metals.

**Figure 3:**
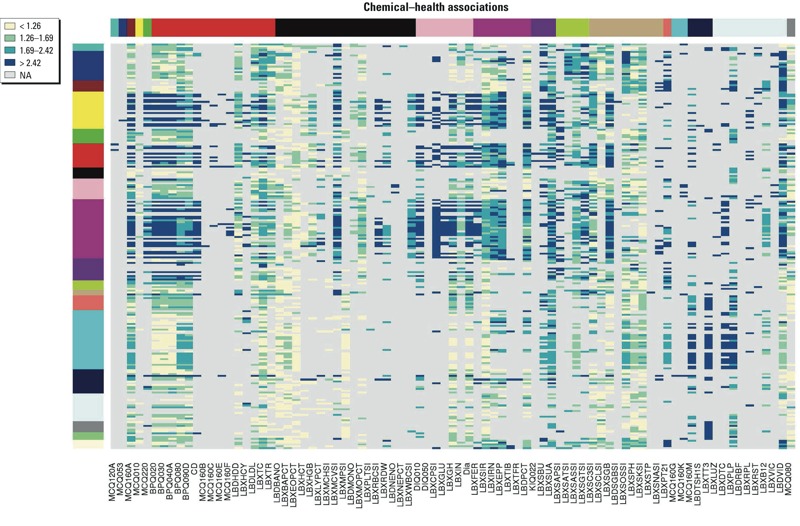
Odds ratios for rules generated from all data (first column from Figure 2). Row color labels indicate the chemical groupings as in Figure 2, and column labels indicated groupings for the health variables. Gray indicates no rule present for the data set (NA). Column colors (top) from left to right: turquoise = allergies; blue = anemia; brown = arthritis; yellow = asthma; green = cancer; red = cardiovascular health; black = complete blood count; pink = diabetes; magenta = iron; purple = kidney; green yellow = liver; tan = multiple associations; salmon = parathyroid; cyan = respiratory; midnight blue = thyroid; light cyan = vitamins and minerals; gray = weight.

*General data properties*. Individual biomarkers of environmental chemicals and disease rates showed some changes across NHANES cycles (see Supplemental Material, Figure S1). Environmental chemicals (see Supplemental Material, Figure S1A–C), generally show a slight decrease in recent cycles in the average amount per individual with notable exceptions (e.g., enterodiol; see Supplemental Material, Figure S1A). Disease prevalence (based on survey responses) tended to increase during this same period (see Supplemental Material, Figure S1D–F). The number of health outcomes associated with a given environmental chemical tended to decrease in later cycles (see Supplemental Material, Figure S2). Figure 2 illustrates the rules generated using the different data stratifications (sex and race/ethnicity), highlighting the diversity of the different strata. Considering only the associations generated from all data (Figure 3), we note that some markers for health are more strongly and commonly associated with high levels of environmental chemicals (for example, vitamin D levels, LBDVID), whereas other markers are associated with very few chemicals (MCQ053, treatment for anemia in previous 3 months) (see Supplemental Material, File S1).

*Prioritization of chemical* → *health associations via FIM*. A list of 7,848 associations between 219 chemicals and 93 markers of health (combining questionnaire and health biomarkers) was generated using all data from 1999–2010 and data strata representing sources of known confounding factors, sex and race/ethnicity (see Supplemental Material, Files S3 and S4, Worksheet “RulesAcrossVarq=0.025conf=0.1su”). Confidence values describe the proportion of associations containing the chemical that also contain the health marker noted and can be used to rank observations for a given chemical. Odds ratios provide a measure of the odds that the chemical and the health outcome are related versus the odds that they are independent. These can be used to compare within and across chemicals within the same association set.

**Figure 4:**
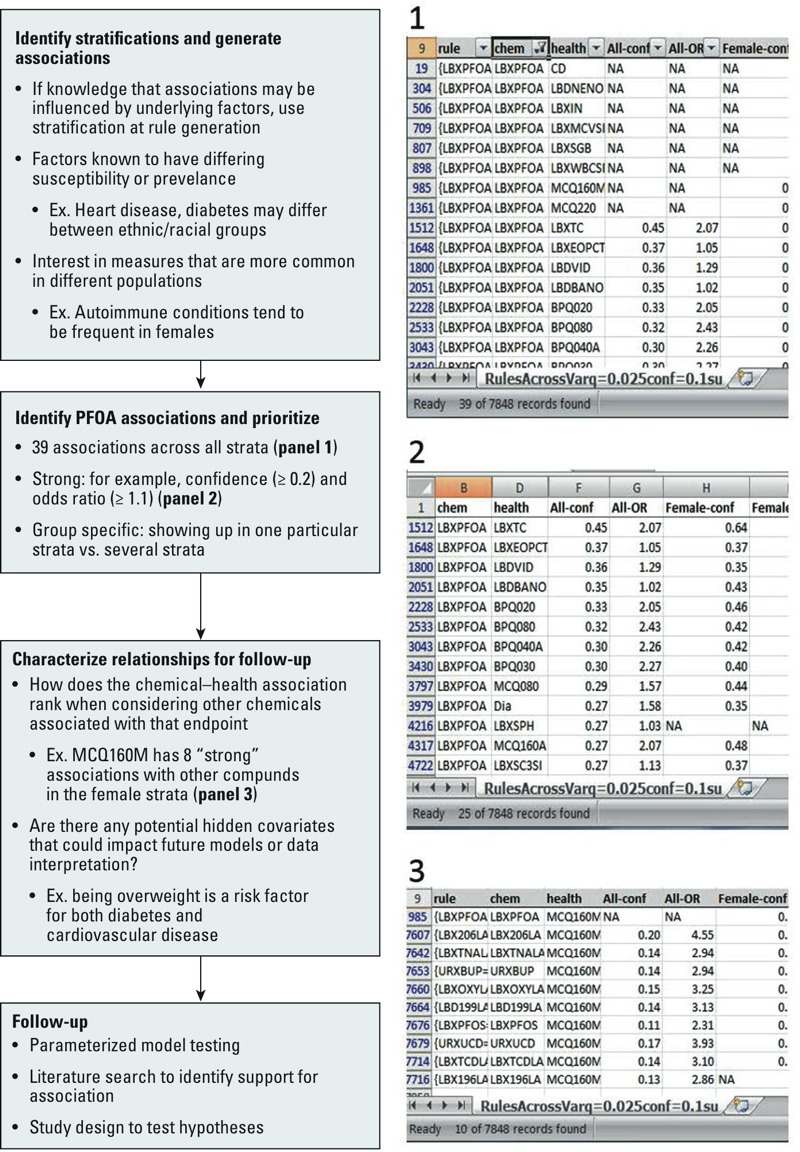
Sample workflow using PFOA. The general workflow for data processing (Figure 1) was employed leveraging additional data strata to obtain a set of rules. Screenshots are generated using Supplemental Material, File S4 (“Spreadsheet”). Ex., example. Rules containing PFOA were extracted (panel 1) then those meeting criteria for strong associations were filtered out (panel 2). Use of additional filters (panel 3) helps to identify and prioritize the relationships for further follow-up.

*Workflow example using PFOA to identify candidates for follow-up*. To illustrate use of the FIM approach for hypothesis generation and prioritization, we present an example using PFOA. All associations generated across the strata are available in Supplemental Material, Files S3 and S4 (“Spreadsheet”). Following the workflow outlined in Figure 1, rules were generated using different data stratifications. All associations with PFOA were extracted from the combined data and across each stratification (sex and race/ethnicity) (Figure 4, panel 1). Initial stratification based on sex and race/ethnicity helps target groups that could have differential disease prevalence for outcomes of interest. Associations with a confidence ≥ 0.2 and an odds ratio ≥ 1.1 were prioritized as “strong” associations (Figure 4, panel 2; see also Supplemental Material, File S4, Worksheet “StrongPFOA”).

In viewing the results, findings that are consistent among several strata are considered top candidates for follow-up either by a targeted literature search or by more quantitative modeling. These include cholesterol and hypertension along with arthritis and overweight. The second group of candidates for further investigation are those with clear differences among the different subgroups such as self-reported thyroid problems in females (MCQ160M) and altered phosphorous (LBXSPH) and uric acid (LBXSUA) seen in the male and non-Hispanic black groups. Characterization of the associations is an important part of the work flow. Here one looks at the co-associations with other chemicals–health outcomes as ways to determine additional support (multiple associations with variables measuring similar states) or possible confounding variables. For example, no clinical markers for thyroid disruption are also associated with PFOA, yet there are several clinical markers to accompany the self-reported markers for high cholesterol; this suggests that the overall weight of evidence for the cholesterol association with PFOA is higher than the thyroid association. In looking at the other chemicals associated with self-reported thyroid problems in the female stratification, there is also an absence of clinical markers. Furthermore, when one looks at the compounds associated with self-reported thyroid problems [PCBs (polychlorinated biphenyls), organochlorine (OC) pesticides, PFOA and PFOS (perfluorooctanesulfonic acid), TCDD (2,3,7,8-tetrachlorodibenzo-*p*-dioxin), and cadmium], there is a reasonable basis for co-exposure (Figure 4, panel 3; see also Supplemental Material, File S4, Worksheet “StrongMCQ160M”). Future models looking at the interaction of PFOA and thyroid should account for these compounds. Similarly the association between PFOA and phosphorous and uric acid in the male and non-Hispanic black strata could result from an underlying relationship with diabetes or hyperparathyroidism.

*Comparison of PFOA associations with the C8 panel findings*. To assess the ability of the FIM method to identify associations, we compared the C8 Science Panel’s (2005) findings on probable link evaluations with PFOA. The C8 Science Panel is a group of public health scientists commissioned to assess whether or not there was support for a probable link between exposure to PFOA and various health outcomes as part of a class action settlement. As part of their work, they did an extensive research review to compile what was known about health effects of PFOA as well as to design and implement new research on exposure effects using a community in the Mid-Ohio Valley whose exposure to PFOA triggered the lawsuit.

Table 1 shows the results of the analysis. NHANES variable labels used for the comparison are shown in the NHANES variable column. Health outcomes studies by the C8 panel were omitted if there was no comparable information from the NHANES subset considered in this paper. Two diseases showed a disagreement between FIM and the C8 panel. For high blood pressure, the measurements used in the studies considered by the panel were different from those used in the NHANES study, and this likely contributed to the disagreement. For arthritis, the panel concluded that there was no trend with increasing exposure. Interestingly enough, there was an association with osteoarthritis and low doses of PFOA noted in the C8 study population. This suggests a possible relationship at lower exposures.

**Table 1:** Comparison of FIM results to the C8 panel findings.

Disease	C8 finding of probable link	FIM association	NHANES variable^*a*^
High blood pressure	No	Yes	BPQ020 BPQ030 BPQ040A
High cholesterol	Yes	Yes	BPQ080 BPQ090D
Coronary heart disease	No	No	CDQ99, MCQ160B-F
Kidney	No	No	KIQ022
Liver	No	No	MCQ160L
Osteoarthritis and rheumatoid	No	Yes	MCQ160A
Asthma	No	No	MCQ010
Thyroid	Yes	Yes	MCQ160M
Cancer	Yes^*b*^	Yes	MCQ220
Type II diabetes	No	No	DIQ010
^***a***^NHANES variables that best matched the end points considered by the C8 panel were used for comparison. Full descriptions of these variables can be found in Supplemental Material, File S1, except for CDQ99 (see “Methods”). ^***b***^Kidney and testicular cancer only.			

*Comparison of diabetes associations with the EWAS findings*. Whereas comparison of the results from the C8 Science Panel (2005) with our FIM approach highlights the distinctions between a thorough literature review and a survey of NHANES, the original EWAS study by Patel et al. (2010) is the closest match to our method in terms of an unsupervised mining of the NHANES data. A comparison of these results shows that a complete survey of the NHANES data using the FIM method compares favorably with a more targeted mining of data for a specific disease outcome via the EWAS approach.

Table 2 shows the number of chemicals in each group that had associations with diabetes markers. The values for the EWAS study are the maximum across all cycles they considered (see “Methods”), whereas the FIM used data from all cycles (1999–2010) in identifying the associations. FIM (fasting blood glucose) highlights the number of associations for each group of environmental chemicals containing elevated blood glucose levels, which matches the criteria used by Patel et al. (2010). The FIM approach picks up slightly more associations for PCBs and OC pesticides, whereas the EWAS study uniquely identifies two heavy metal associations. When comparing the specific chemicals, our method detected 11 of the 18 chemicals (61%) from the EWAS study along with 16 not previously identified. Of the 7 chemicals missed by our method, the 5 nonmetals do not have data for cycle years later than 2004. The diabetes marker was measured in all cycle years and the apparent prevalence has increased in later years (see Supplemental Material, Figure S1F); this artificially reduces the associations seen across all cycle years because there is no opportunity for co-occurrence. The stronger associations from the EWAS study, including the two chemicals identified in two separate cycle years, were still detectable in our analysis despite this fact. This demonstrates that for strong associations the method is robust to missing data.

**Table 2:** Comparison of the number of associations found for diabetes.

Group	EWAS: maximum across 99-06 cycles	FIM^*a*^: fasting blood glucose (direct matches with EWAS)	FIM^*b*^: diabetes marker (direct matches with EWAS)
Organochlorine pesticides	2	5 (1)	8 (2)
Polychlorinated biphenyls	11	19 (9)	24 (10)
Heavy metals	2	0	23 (2)
Dioxins and furans	3	3 (1)	13 (2)
Volatile compounds	0	0	7
Other pesticides	0	0	1
Phenols	0	0	9
Phthalates	0	0	6
Dialkyls	0	0	1
Hydrocarbons	0	0	9
Perchlorate	0	0	1
Polyflouro-chemicals	0	0	5
^***a***^Total number of associations for LBXGLU using FIM across 1999–2010. ^***b***^Total number of associations for Dia using FIM across 1999–2010.			

An additional FIM metric (diabetes marker, Dia) considers high hemoglobin A1C or whether the subject reported being diagnosed as a diabetic in addition to the blood glucose measurement. Using this variable increased the total number of associations from 27 to 107 (Table 2). For a broad survey of this nature with very sparse responses, this aggregate marker helps to capture lower-strength associations. For example, all but two of the chemicals from the Patel study (Patel et al. 2010) are identified using the FIM method when this more inclusive diabetes metric is used. Because the goal of this work is to identify possible associations for more detailed follow-up, the argument for using a more restrictive marker such as glucose seems less compelling. However, because our results include both metrics, along with the other individual components included in the combined marker, this decision can be left to the user.

## Discussion

*Findings from the case studies*. In both the C8 panel comparison and the EWAS comparison, most of the relationships identified were recovered using the FIM approach. In the first case study (Table 1), discrepancies were attributable to the evidence under consideration by the C8 panel. In the case of high blood pressure, the precise measurements used were different, and the choice of which measurement is most appropriate should be made in the context of the specific question being asked. In the case of arthritis, the expert panel considered dose trends, which are a key consideration when determining causality. This case study highlights that broad surveys such as the FIM mining of the NHANES data presented here should never be considered a replacement for an expert review of the scientific literature. The data presented, however, could provide an ideal starting point for such a panel because all of the diseases identified by the panel were flagged in the FIM survey.

The second case study demonstrated that the results from this comprehensive survey of NHANES are comparable with a more targeted study focused on a single disease end point. The slight increase in findings for FIM compared with EWAS for comparable markers (Table 2) was not surprising given the additional cycles investigated and potentially less stringent criteria due to the intentionally low confidence level used in FIM. The use of an aggregate marker such as the diabetes marker (Table 2), which is less sensitive to variability of spot measurements in individual variables, captures many more potential associations than either method using a single marker; but prudent follow-up should include consideration of the associations seen with each marker individually. Together, these results suggest that the results from the FIM represent an ideal starting point for either evaluating diseases potentially associated with a given chemical or chemicals that may be associated with a given disease. By providing a comprehensive list of associations from NHANES, the relative strength of association can be considered rather than attempting to interpret *p*-values in light of the extensive multiple testing inherent with this data set (Sobus et al. 2015).

In general, it appears that the changing prevalence of both disease and volume of environmental chemicals had an effect on the associations found for the different cycles (see Supplemental Material, Figures S1 and S2). Using set criteria instead of a distribution-based cutoff for identifying “presence” of the environmental chemical or using a range of cutoffs for determining when an individual is “exposed” (Bell and Edwards 2014) may be desirable to adjust the false nondiscovery rate (Genovese and Wasserman 2002; Sarkar 2002; Storey 2003). For example, serum lead < 20 μg/dL in adults is considered to be within the threshold for a clinically normal range (MedlinePlus 2013), though acceptable levels in children are well below this value. The cutoff for the NHANES variable used in the analysis (LBXBPB) was 6.02 μg/dL (see Supplemental Material, File S2), which was based on the population distribution. Most of the NHANES individuals used in this analysis had levels far below this (see Supplemental Material, Figure S1), and the median level declined each cycle. As a result, use of the population distribution for each cycle independently would result in different cutoffs over time with potentially drastic impacts on the associations seen when looking at 1999–2000 cycle versus the 2009–2010 cycle. The aggregate analysis using the full population across all cycle years to establish “exposed” or a fixed level is likely to decrease this effect.

Figure 2 shows there is good overall correlation between the rules generated using all the subgroups and those where subsets based on sex or race were used; however, it also highlights associations that are obtained only by looking at a subset of the data. This suggests that depending on the relationship or populations of interest, it may be desirable to consider associations mined from these subsets separately, as recommended in the workflow (Figures 1 and 4). The association table (see Supplemental Material, Files S3 and S4 (Workbook “RulesAcrossVarq=0.025conf=0.1su”) generated using the FIM approach easily facilitates such additional considerations as shown in Figure 4. Further subgroups of interest can be easily incorporated into the workflow for consideration.

*Advantage of using FIM to prioritize and control for multiple testing*. One use of the proposed method is to aid in prioritization of associations. For example, when evaluating several separate studies all linking a single health outcome to multiple chemicals, these results can provide a common association metric for comparison among the chemicals. When one is evaluating a single report of a chemical association with a health outcome, these results can provide an indication of how the strength of that association compares with other chemicals and/or what other chemicals might need to be considered in a cumulative risk context. Alternatively, these results can be used prospectively to identify putative chemical–outcome relationships or possible co-occurring compounds for more detailed analysis.

Using the EWAS approach, Patel et al. (2010, 2012a, 2012b) presented a strategy to associate environmental factors from the NHANES surveys to a specific disease. Using multiple logistic regression models and controlling for multiple testing, they were able to generate a list of statistically significant associations between the environmental chemicals and a health measure, such as the type 2 diabetes example described here (T2D, defined as fasting serum glucose ≥ 126 mg/dL) (Patel et al. 2010). This approach has distinct advantages over more ad hoc approaches: They can compare multiple relationships; and the model is relatively transparent with respect to why given relationships were considered. The FIM strategy extends this further by investigating all chemical–health relationships simultaneously with relatively little computational overhead. Thus, for a given chemical–health relationship, one can identify not only other chemicals that possibly relate to that health state, but also other health states related to each chemical along with the relative strength of association as described in the example workflow. Because both multiple regression models and FIM have distinct advantages and disadvantages, as discussed below, they should be considered complementary approaches to a complex problem.

Most EWAS studies reported previously have used some form of regression model. These models fit into a hypothesis-testing framework allowing for specific calculation of type 1 and type 2 error and can control for confounding variables (e.g., sex, race/ethnicity, smoking status). The FIM method (Bell and Edwards 2014) provides a comprehensive description of relationships for the entire data set, providing the information needed for generating hypotheses. Although it does not facilitate control over confounding variables aside from data stratification, it does comprehensively report all associated variables that were included in the study. This could be used in conjunction with more traditional regression models to avoid missing potential confounders, as suggested in the follow-up options provided in Figures 1 and 4. The NHANES study design results in variables that are never measured together in an individual (e.g., some urinary chemicals). These are impossible to combine in a regression model. The FIM method cannot give any information on the interactions among these variables; however, it can be used to identify possible confounding chemicals/diseases even if the data set does not include any co-occurring measures.

Furthermore, the FIM method enables a quick relative comparison with other measures on either side of the relationship. Comparing the confidence values, one can easily prioritize the health outcomes most likely associated with a particular chemical. Using odds ratios, one can see how likely an outcome is to be associated with other chemicals. The FIM can also be used to extract chemical–chemical or health–health associations just as the chemical–health associations are extracted. This can give better insight to highly related variables within the data set, indicating possible common co-exposures or redundant markers (Bell and Edwards 2014).

Follow-up of the associations is a key part of the workflow because an association is only describing the data set. If using the associations to prioritize or put into context literature findings, then the ranked lists are sufficient. If the end goal is hypothesis building, then the associations can be used to help guide a structured literature search and provide guidance in properly parameterizing a model to decrease multiple testing. One benefit of using association rules is that it is very quick and easy to obtain relationships between various chemicals and heath markers because the data are already processed. The nature of the rules lends itself nicely to a graphical exploration as well which can be helpful in integrating other information sources in the hypothesis-building phase (Bell and Edwards 2014).

## Conclusions

As demonstrated, the FIM approach enables prioritization and comparison of associations found in the NHANES data set. The list of chemical–health associations can be used to identify those health metrics that are most likely to occur for a given chemical as well as the chemicals most likely to associate with a given health metric. This allows prioritization of follow-up studies to evaluate possible causal relationships. Because the method is computationally efficient (< 30 min on a standard laptop), it allows for a comprehensive analysis of all chemicals and health metrics, not just a subset of chemicals or a single health outcome. This allows the resulting list to be used for the evaluation of previously published associations because they can now be compared with all other associations for the chemical–health outcome in question. We expect that this approach can be extended to similar data sets and can provide a framework for researchers and risk managers in interpreting these types of studies.

## Supplemental Material

(311 KB) PDF

(3.8 MB) ZIP
