# Supplementary material for: Identification and Prioritization of Relationships between Environmental Stressors and Adverse Human Health Impacts
Source: Environ Health Perspect. 2015 Apr 10;123(11):1193–9. doi: 10.1289/ehp.1409138 (PMC4629746; doi:10.1289/ehp.1409138)
Supplement: (311 KB) PDF [file ehp.1409138.s001.acco.pdf]

Environ Health Perspect
doi: 10.1289/ehp.1409138

Note to Readers: *EHP* strives to ensure that all journal content is accessible to all readers. However, some figures and Supplemental Material published in *EHP* articles may not conform to 508 standards due to the complexity of the information being presented. If you need assistance accessing journal content, please contact ehp508@niehs.nih.gov. Our staff will work with you to assess and meet your accessibility needs within 3 working days.

Supplemental Material

Identification and Prioritization of Relationships between Environmental Stressors and Adverse Human Health Impacts

Shannon M. Bell and Stephen W. Edwards

Table of Contents

Figure S1. Changes in response across cycles Select biomarker levels (A-C) and disease rates (D-F) based on questionnaire responses for each NHANES cycle. X axis indicates the NHANES cycle (y9900 = cycle years 1999-2000, etc. In box plots (A-C), boxes extend from the 25th to the 75th percentile, horizontal bars represent the median, whiskers extend 1.5 times the length of the interquartile range (IQR) above and below the 75th and 25th percentiles, respectively, and outliers are represented as points.

Figure S2. Rules across NHANES cycles. Odds ratios of rules across all cycles considered (All) or individual cycle years (given in column labels). Color labels on the left highlight the different chemical groups for the association. From bottom to top: Light yellow=drinking water volatile organic compounds, light green= urinary perchlorate, nitrate, and thiocyanate, grey = phytoestrogens, light cyan = phthalates, midnight blue: urinary metals, cyan = current use pesticides, salmon= urinary arsenic, tan = smoking, green yellow = environmental pesticides, purple = PFC, magenta = PCB, pink = PAH, black = organophosphate pesticides, red = organochlorine pesticides, green = environmental phenols, yellow = DFP, brown= carbamates, blue = blood volatile organic compounds, turquoise = blood metals.

Additional Files

File S1. NHANES Variables Used in This Study. This table is available as a tab delimited file. Variable.Name: the variable names from NHANES (or described in this paper) used as the identifier. Variable.Description: short description of the variable. Health: the type of marker (E,H,Q) that the measure represents. Group: what grouping (for the purpose of analysis) the measure was assigned to. Begin.Year and End.Year columns contain a year if the variable was measured that cycle year and an NA otherwise.

File S2. Environmental chemical and health variable summaries and parameters used for discretization. Values are from the distribution of a variable across all cycles considered. Label: the NHANES label. Min= the minimum value, 25% = value at the 25%ile, median= median value, 75% = value at the 75%ile, max= the maximum value, upper = the upper range used in discretization, values greater than this were assigned a "1", lower= the lower range used in discretization, values below this were assigned a "-1", source= the source used to discretize. For Source, "population based" means that the population distribution was used to determine the high and or low values, a url reference is a reference to the source used for determining upper or lower limits. If there was no upper limit then "1000000" was used in the "upper" column and if no lower limit "-1" was used in the lower column. For more details refer to the code available as Supplemental File S5.

File S3. FIM Association Rules. This table is available as a tab delimited file. rule: the association rule identified through frequent itemset mining. chem: the chemical label. Chem.Desc : the chemical description. health: the health (H or Q) label. Health.Desc: the health description. X-conf: the confidence level for the rule using the data from X group of individuals, X-OR: the odds ratio for the rule using data from X group individuals. All = all individuals, Female = all females, Male= all males, MexAm= Mexican Americans, NHBlack = non-hispanic black, and NHWhite= non-hispanic white.

File S4. Workbook of association rules. This file contains all the rules generated using the workflow presented in the manuscript. The association rule (rule) gives the chemical -> health relationship. 1 indicates high or present, -1 indicates low. For example {LBX066LA=1} => {LBDHDD=-1} means that high levels of PCB66 are associated with low HDL cholesterol

Columns "chem" and "health" are the variable names from the NHANES data and columns C and E are the full label name/description. All is all data across the 1999-2010 cycles, Female and Male are those strata. MexAm= Mexican American, NHBlack= non-hispanic black, NHWhite= non-hispanic white. OR refers to the odds ratio and conf the confidence.

To use this file: rows 1:8 are for use in advanced filtering. Items on the same row are filtered using an "AND" relationship, items on different rows are an "OR" relationship. To filter by a variable, for example PFOA, type "LBXPFOA" in the "chem" column and select the advanced filter under the data tab. The list range will show A9:$Q$7857 and the criteria range will be $A$1:$Q$8. To select the strong associations (as shown in Figure 4 from the main manuscript), copy "LBXPFOA" in column B (chem) from rows 2 through 7 and on separate rows have the selection criteria (see "StrongPFOA" sheet). To get the results shown in Figure 4, panel 3 remove the filtering criteria from the "chem" column and add in "MCQ160M" in the "health" column (see "StrongMCQ160M" sheet).

In setting filters on confidence, it is important to remember that the confidence value reflects the proportion of occurances of the chemical for which the health outcome was also present. Thus higher confidence values means that the relationship occurs more frequently. Larger odds ratios implies that it is rare for the health impact to occur if the chemical didn’t, so higher values are generally desirable. Due to simple math though, if a particular health outcome is rare it may simply give a large odds ratio due to a small denominator. We suggest that the both of these values be used together in filtering and prioritizing.

File S5. Code. This folder contains the code and data to repeat the analyses presented in the manuscript. See the file “InstructionsForRunningScripts.txt” inside the compressed folder for execution details.

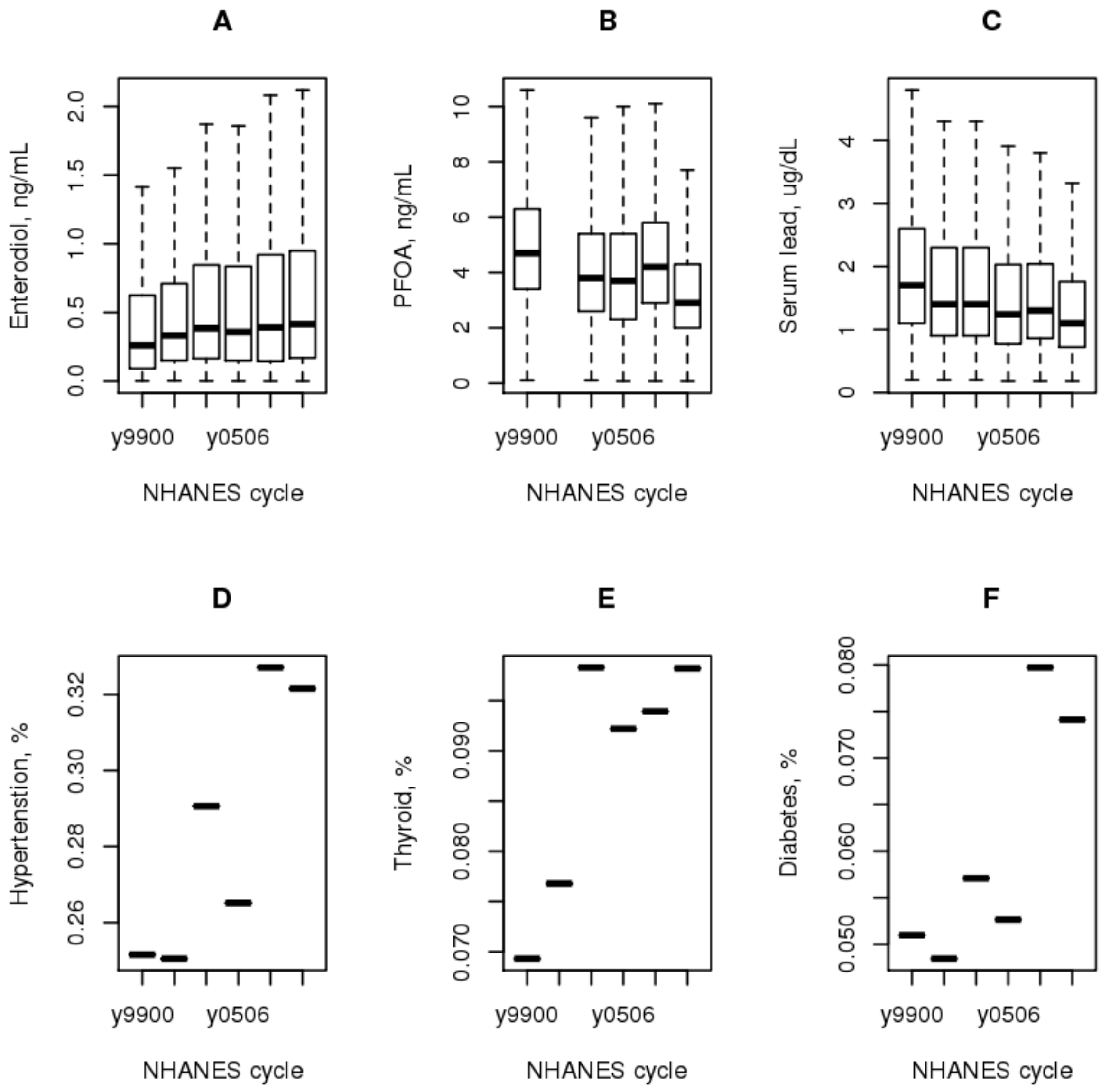

Figure S1. Changes in response across cycles Select biomarker levels (A-C) and disease rates (D-F) based on questionnaire responses for each NHANES cycle. X axis indicates the NHANES cycle (y9900 = cycle years 1999-2000, etc. In box plots (A-C), boxes extend from the 25th to the 75th percentile, horizontal bars represent the median, whiskers extend 1.5 times the length of the interquartile range (IQR) above and below the 75th and 25th percentiles, respectively, and outliers are represented as points.

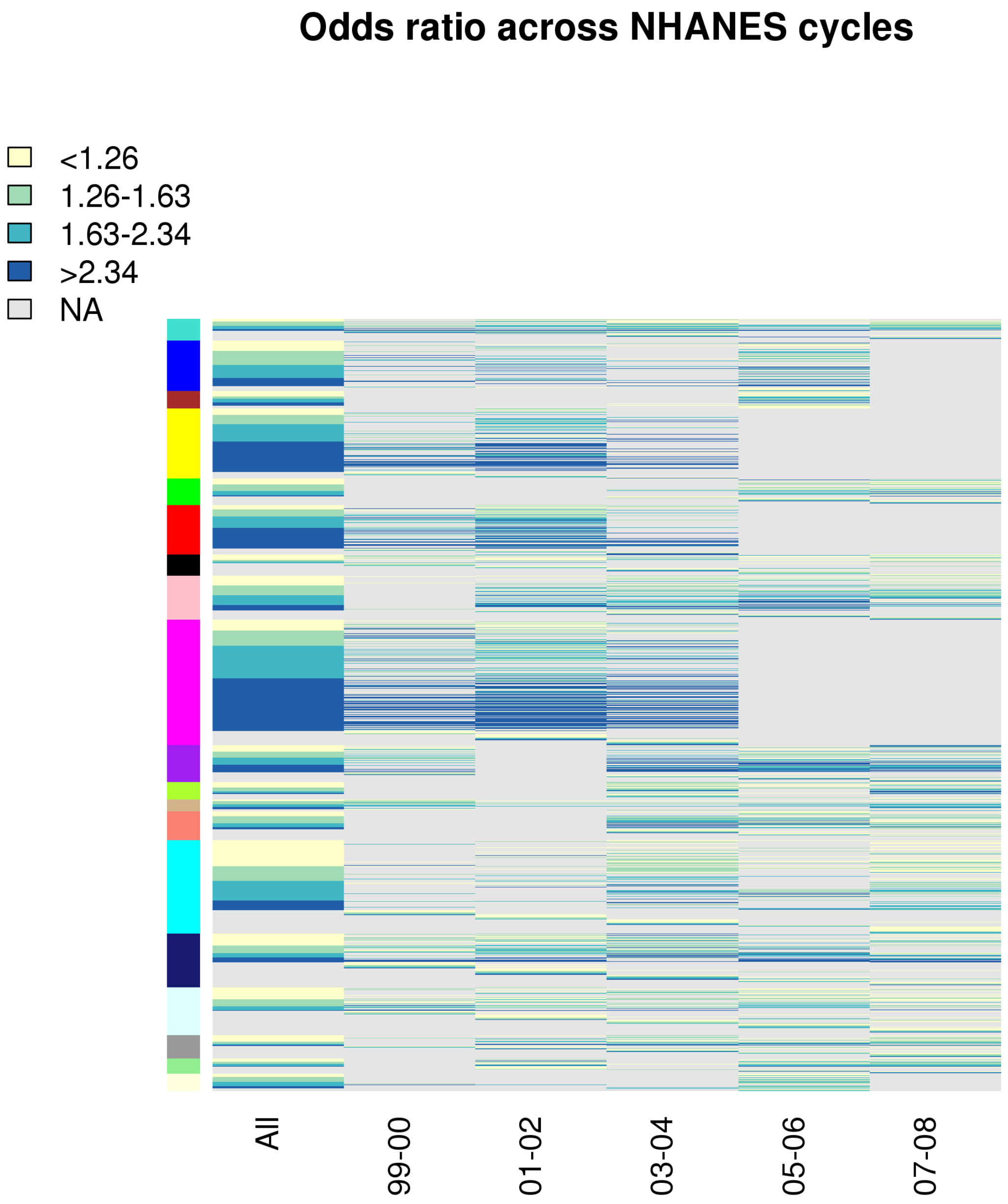

Figure S2. Rules across NHANES cycles. Odds ratios of rules across all cycles considered (All) or individual cycle years (given in column labels). Color labels on the left highlight the different chemical groups for the association. From bottom to top: Light yellow=drinking water volatile organic compounds, light green= urinary perchlorate, nitrate, and thiocyanate, grey = phytoestrogens, light cyan = phthalates, midnight blue: urinary metals, cyan = current use pesticides, salmon= urinary arsenic, tan = smoking, green yellow = environmental pesticides, purple = PFC, magenta = PCB, pink = PAH, black = organophosphate pesticides, red = organochlorine pesticides, green = environmental phenols, yellow = DFP, brown= carbamates, blue = blood volatile organic compounds, turquoise = blood metals.
